# A Fiber-Optic Fluorescence Microscope Using a Consumer-Grade Digital Camera for *In Vivo* Cellular Imaging

**DOI:** 10.1371/journal.pone.0011218

**Published:** 2010-06-23

**Authors:** Dongsuk Shin, Mark C. Pierce, Ann M. Gillenwater, Michelle D. Williams, Rebecca R. Richards-Kortum

**Affiliations:** 1 Department of Bioengineering, Rice University, Houston, Texas, United States of America; 2 Department of Head and Neck Surgery, The University of Texas MD Anderson Cancer Center, Houston, Texas, United States of America; 3 Department of Pathology, The University of Texas MD Anderson Cancer Center, Houston, Texas, United States of America; The University of Queensland, Australia

## Abstract

**Background:**

Early detection is an essential component of cancer management. Unfortunately, visual examination can often be unreliable, and many settings lack the financial capital and infrastructure to operate PET, CT, and MRI systems. Moreover, the infrastructure and expense associated with surgical biopsy and microscopy are a challenge to establishing cancer screening/early detection programs in low-resource settings. Improvements in performance and declining costs have led to the availability of optoelectronic components, which can be used to develop low-cost diagnostic imaging devices for use at the point-of-care. Here, we demonstrate a fiber-optic fluorescence microscope using a consumer-grade camera for *in vivo* cellular imaging.

**Methods:**

The fiber-optic fluorescence microscope includes an LED light, an objective lens, a fiber-optic bundle, and a consumer-grade digital camera. The system was used to image an oral cancer cell line labeled with 0.01% proflavine. A human tissue specimen was imaged following surgical resection, enabling dysplastic and cancerous regions to be evaluated. The oral mucosa of a healthy human subject was imaged *in vivo*, following topical application of 0.01% proflavine.

**Findings:**

The fiber-optic microscope resolved individual nuclei in all specimens and tissues imaged. This capability allowed qualitative and quantitative differences between normal and precancerous or cancerous tissues to be identified. The optical efficiency of the system permitted imaging of the human oral mucosa in real time.

**Conclusion:**

Our results indicate this device as a useful tool to assist in the identification of early neoplastic changes in epithelial tissues. This portable, inexpensive unit may be particularly appropriate for use at the point-of-care in low-resource settings.

## Introduction

Point-of-care diagnostic devices should be small, inexpensive, and portable, yet accurate, robust, and simple to use. Optical imaging techniques can play a crucial role in the realization of such technologies, by providing real-time, high-resolution diagnostic information in non- or minimally-invasive fashion. In addition, flexible, miniature fiber-optic components have made it possible to access tissue at confined sites within the body, enabling cellular level imaging to be performed in tandem with standard wide-field methods, such as endoscopy. These capabilities are currently being investigated for potential roles in clinical diagnostics, screening, and surgical guidance [1]–[9], but for translation of the technology to the point-of-care setting to become realistic, issues of cost, complexity, size, and performance must be addressed.

Rapid improvements in the technical specifications and cost efficiency of consumer-grade electronics have brought high-performance imaging devices to the general market. Low-cost, high-quality digital cameras are now available with over 20 megapixel image sensors (for example the Canon EOS-1Ds mark III); Sony-Ericsson's *Satio* model cellular phone has 12.1 megapixels. SLR (Single Lens Reflex) digital cameras are relatively inexpensive in comparison to the scientific-grade CCD cameras which are used in many biological imaging applications [10]–[12]. Most of these cameras are powered by a rechargeable battery pack and include a built-in LCD screen for real-time visualization. These features can support the design of imaging systems that are low-cost, battery-powered and completely portable. Indeed, studies have employed digital SLR cameras for macroscopic image acquisition of biological tissues [13]–[16], and also for recording images of cells and tissue sections on conventional and portable microscopes [17].

Microscopic scale imaging *in vivo* has thus far been developed through techniques such as confocal microscopy, using flexible, narrow fiber-optic probes to access superficial tissues such as the skin, or hollow cavities such as the oral cavity, bronchus, cervix or GI tract [1]–[6]. While these systems have demonstrated the capacity to provide high-quality images, the requirements of laser sources, scanning mechanism(s), and high-speed digitizing hardware all contribute to a price tag well out of the range of many healthcare settings. Our group [2], [5], and others [1], [7], [9] have recently demonstrated sub-cellular resolution wide-field imaging through a fiber-optic bundle. By using a wide-field epi-fluorescence arrangement instead of point-scanning, the system complexity and cost are greatly reduced. When used with bright, fluorescent contrast agents, sub-cellular morphology can be viewed in real-time, by simply placing the distal end of the bundle onto the tissue site to be imaged.

Here we present a high-resolution fiber-optic fluorescence imaging system using a consumer-based digital camera to visualize sub-cellular features in living tissue. We demonstrate the capabilities of the system through a series of experimental studies. First, we carried out imaging of a cultured cell model of an oral cancer cell line labeled with fluorescent dye. Next, we performed imaging of a surgically-resected human tissue specimen, including dysplastic and cancerous regions. Finally, a healthy human subject was imaged *in vivo*. These studies demonstrate the capability of the system to obtain images with sub-cellular resolution, non-invasively, and in real-time. We propose that this portable, inexpensive diagnostic imaging device may be useful as an efficient diagnostic tool at the point-of-care for populations in remote or rural communities in the U.S. as well as in developing countries.

## Materials and Methods

### Fiber-optic microendoscope system using a consumer-grade digital camera

**Figure 1:**
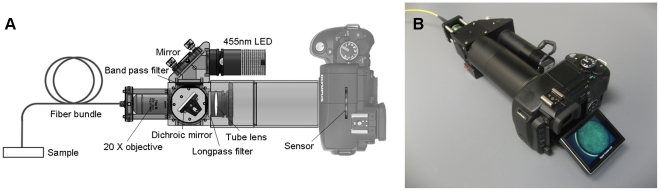
High-resolution fiber-optic microendoscope. (A) Schematic diagram of the system. (B) Photograph of the system.

The main components of the system include an LED light source, a microscope objective lens, a fiber bundle and a digital camera as shown in Figure 1. The LED light source emits an optical spectrum centered at 455 nm with 20 nm spectral bandwidth (full-width half-maximum). Following a 450 nm bandpass filter (Thorlabs, FB450-40) and a 475 nm dichroic mirror (Chroma, 475DCXRU), excitation light illuminates the proximal end of a 1 mm diameter coherent fiber-optic bundle (Sumitomo, IGN-08/30). The distal end of the bundle is placed in direct contact with the sample to collect fluorescence emission, which then returns through the bundle and is imaged on to the optical sensor of the digital camera by a 20×/0.40 NA infinity-corrected objective lens (Olympus) and a 150 mm tube lens. For proflavine (Sigma, P2580) used as a contrast agent, a 500 nm long-pass filter (Thorlabs, FEL0500) was placed in infinity space. The objective and the tube lens combination form a magnified image of the bundle on the sensor of the camera, which is visualized on the LCD screen of the camera in real-time. The camera also allows connection to a laptop or other monitor screen through USB or composite video cables. The entire system weighs 3.5 pounds, is powered by a rechargeable battery, and operates for about one hour on a single charge. The overall cost of the system is about $2,000 including the $400 digital SLR camera body. The SLR camera specifications are summarized and compared with those of the scientific-grade CCD camera used by Muldoon *et al.* [2] in Table 1.

**Table 1:** Experimental parameters of systems.

	Olympus E-330	Muldoon *et al.* [2]
Array size (mm)	17.3×13.0	10.2×8.3
Pixel size (µm)	5.6×5.6	6.45×6.45
Number of pixels	3136×2352	1392×1040
Relay magnification	19.5×	8.3×
Pixels per fiber at CCD	7.68	2.85
CCD dynamic range	43 dB	67 dB

### Cell culture and labeling

Proflavine is a fluorescent stain which labels cell nuclei by intercalating between DNA base pairs [18]. Absorption and emission maxima are at approximately 445 and 510 nm, respectively. 1483 oral cancer cells derived from a human oropharyngeal squamous carcinoma were stained with proflavine (0.01% w/v in PBS, Sigma P2508) and then suspended in collagen for imaging.

### Surgical specimen acquisition and imaging

Through a study protocol approved by both Rice University and the University of Texas M.D. Anderson Cancer Center Institutional Review Boards, and following written informed consent by the patient, a surgical specimen was obtained immediately after resection. Following topical application of proflavine (0.01% w/v in PBS, Sigma P2508) to the mucosal surface, images were obtained with the fiber-optic microendoscope. The specimen was sent for routine histopathology; H&E sections were prepared, including from the sites imaged with the fiber-optic microendoscope. Proflavine staining does not affect subsequent H&E staining for histologic analysis.

### Human subject imaging

The oral mucosa of a healthy human subject who had given written informed consent was imaged *in vivo* using the fiber-optic microendoscope in accordance with a protocol approved by the Rice University Institutional Review Board. The participant in this manuscript has given written informed consent (as outlined in the PLoS consent form) to publication of his/her case details. Proflavine was obtained in powder form from Sigma (P2508) and prepared in solution for imaging by dissolving in PBS (0.01% w/v) and sterile filtered prior to use. Proflavine was topically applied to a small area of the mucosal surface. After only a few seconds of application, the distal tip of the fiber-optic bundle was placed in direct contact. Real-time observation of sub-cellular detail at the imaged site was possible via the camera's LCD screen (Figure 1 (B)). Images recorded for additional analysis were stored on the camera's removable memory card.

## Results

### System characterization

**Figure 2:**
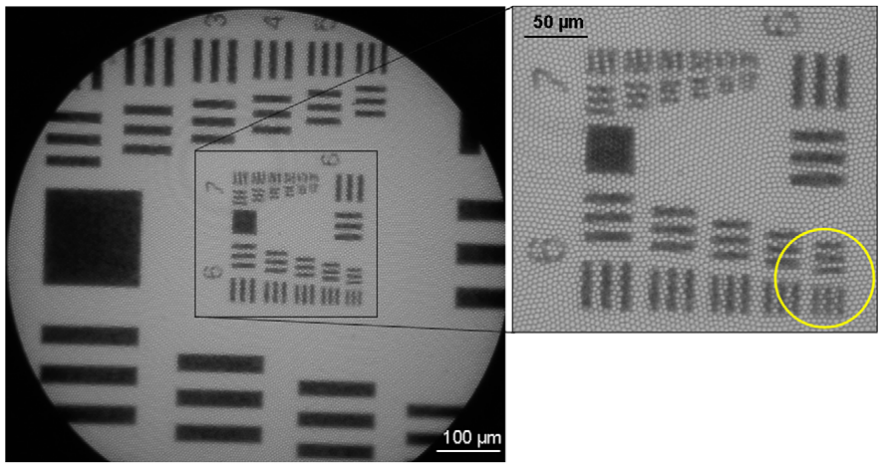
USAF resolution target image.

Spatial resolution was measured by imaging a Ronchi grating and calculating the distance across the edge over which intensity ranged from 10% to 90% of the maximal value. The 10–90% distance was found to be 5.0 µm. The spatial resolution is currently limited by under-sampling due to the 4 µm core-core spacing between individual elements in the coherent fiber-optic bundle; Figure 2 shows that the system can resolve the G6 E6 lines of a USAF resolution target (line width = 4.4 µm). The size of the individual fibers is 2.2 µm and there are approximately 30,000 fibers in the bundle. We assessed the depth-of-field of the system by measuring images of a USAF resolution target as the distance between the fiber tip and the surface of the target was increased. Results show that the depth of focus is approximately 20 µm, based on the distance at which the contrast between the G6 E6 lines was reduced to 26% of its maximal value (Rayleigh). The imaged field-of-view corresponds to the physical area of the fiber bundle face, which is 800 µm in diameter. In the system presented here, the fiber-bundle image slightly overfills the sensor of the camera, resulting in an achieved field-of-view of 660 µm. The optical power delivered to the distal tip of the fiber bundle was measured to be 0.5 mW, corresponding to an average irradiance level of 100 mW/cm^2^.

### 1483 oral cancer cell imaging

**Figure 3:**
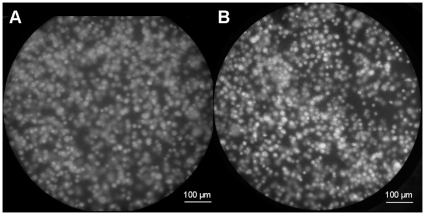
1483 oral cancer cell images using proflavine as a contrast agent to visualize cell nuclei. (A) Image acquired with the SLR-based microendoscope. (B) Image acquired with the scientific CCD-based microendoscope.

Images of 1483 oral cancer cells labeled with proflavine obtained using the fiber-optic microendoscope are shown in Figure 3. Owing to labeling with proflavine, cell nuclei appear bright in the image. Images acquired with the SLR-based system (Figure 3 (A)) were compared to those obtained with a scientific CCD-based microendoscope (Figure 3 (B)) [2], with both images acquired at the same sample site, under identical illumination conditions. The power measured from the fiber bundle was 0.5 mW for each image. We summarized the experimental parameters of these two microendoscope systems in Table 1. The SLR-based image (Figure 3 (A)) was taken with a 1 second integration time at the lowest ISO setting (ISO 100). The CCD-based image (Figure 3 (B)) was taken with a 1 second integration time at 0 dB gain. Each image was separated into labeled (signal) and unlabeled (background) regions in software by setting an intensity-based threshold. The average grayscale intensity of the pixels in the labeled regions in the SLR-based image was 80.4. The corresponding average intensity in the CCD-based image was 138.9. The signal-to-background ratio measured in the SLR-based image was 2.88 and 3.67 in the CCD-based image. The average pixel intensity of the black level measured in the SLR-based system was 2.3 and 5.6 in the CCD-based system; it is not possible to manually adjust the offset level in either system.

### *Ex vivo* human specimen imaging

**Figure 4:**
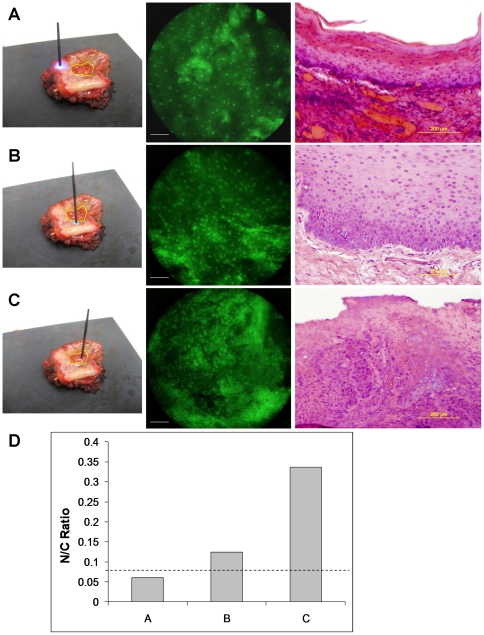
Ex vivo human specimen imaging. (A) Normal epithelium. Left; photograph of fiber bundle probe in contact with resected tissue at clinically-normal region. Yellow border represents margin of clinically-abnormal region identified by the surgeon. Center; image taken with fiber-optic microendoscope. Scale bar represents 100µm. Light; corresponding histopathology section demonstrating normal epithelium. (B) Mild dysplasia. Probe placed at region near to margin of tumor (left). Corresponding histopathology section demonstrates mild dysplasia. (C) Cancer. Probe placed at clinically-abnormal region. Corresponding histopathology section demonstrates squamous carcinoma. (D) Calculated N/C ratio of images in (A, B and C). The dashed line represents an N/C ratio of 0.08.

Images obtained from a surgically resected oral tissue specimen containing an oral squamous carcinoma located in the right posterior floor of mouth are shown in Figure 4. Each image was acquired with a 0.25 s integration time and ISO 400. The distal tip of the fiber-optic bundle was placed in direct contact with the specimen at regions appearing clinically normal and abnormal, as shown in the accompanying photographs. In microendoscope images, regularly-distributed nuclei appear as discrete, bright dots throughout the field-of-view at the clinically-normal region (Figure 4 (A)). Near the tumor margin, the nuclear density exhibited the characteristic increase associated with neoplastic progression (Figure 4 (B)). The image obtained at the region containing the tumor demonstrates the characteristic features associated with cancer such as dense and disorganized nuclei (Figure 4 (C)). From the microendoscope images, we quantified the nuclear-to-cytoplasmic (N/C) area ratio, which is an important parameter used in the histological diagnosis of cancer. Using morphological image processing methods, nuclei were segmented based on a pixel intensity threshold, defining nuclear and cytoplasmic regions. N/C ratio was then calculated from the number of pixels in each region. The N/C ratio for the images shown in Figure 4 (A, B and C) was calculated to be 0.06, 0.12 and 0.34 respectively (Figure 4 (D)), which may be compared to the threshold value of 0.08, established by Collier *et al.* as a means of discriminating between normal (lower N/C ratio) and cervical intraepithelial neoplasia (higher N/C ratio) [19]. The corresponding histology in Figure 4 (A) demonstrates normal epithelium, while Figure 4 (B) indicates mild dysplasia and Figure 4 (C) indicates squamous carcinoma.

### *In vivo* human subject imaging

**Figure 5:**
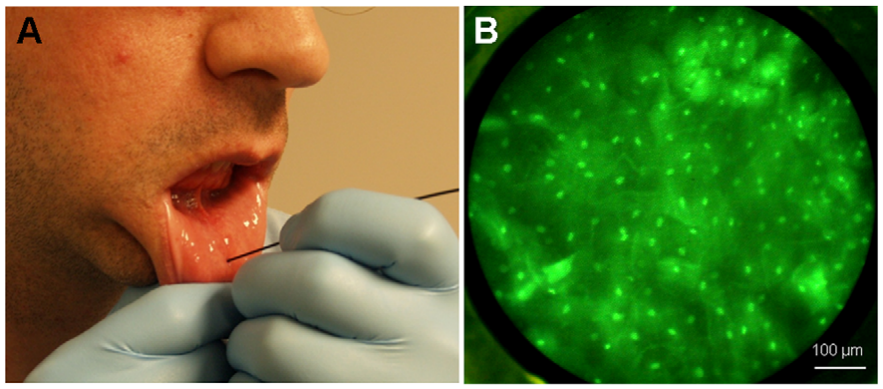
In vivo image of human volunteer.

Figure 5 demonstrates the characteristics of normal human oral mucosa imaged *in vivo*, including bright and regularly-distributed nuclei labeled by proflavine. The calculated N/C ratio for this image is 0.05. The image presented in Figure 5 (B) is a single frame acquired during real-time imaging at 4 frames per second.

## Discussion

Several studies have previously investigated the use of compact digital cameras or cell phone cameras for biomedical imaging applications. Consumer-grade cameras have been used for wide-field fluorescence imaging [13]–[16], pathology analysis [17], and the on-board cameras of cell phones [20]–[24] and PDAs [25] have been used in clinical applications. Here we demonstrated a portable high-resolution fiber-optic fluorescence imaging system for *in vivo* cellular imaging. The system leverages the increasing level of imaging performance which has become accessible at relatively low-cost through the consumer electronics market. The use of a digital camera has several practical benefits; the system incorporates an LCD screen for real-time image visualization, thus eliminating the requirement for additional hardware for image processing and display. While digital camera displays have increased in size and resolution in recent years, many cameras can also transmit images to a larger external monitor via USB or composite video cables. Digital camera units are also battery-powered, enabling a completely portable imaging system to be assembled.

The images obtained here were obtained using the contrast agent, proflavine. Proflavine is inexpensive, has a long history of safe clinical use, and fluorescence images of epithelial cells can be obtained with high contrast between the cytoplasm and nucleus within seconds after topical application. Proflavine is the principal component of acriflavine and has been used for fluorescence imaging in the European, Asian, and Australian gastrointestinal literature without any adverse effects noted [26]. Moreover, proflavine has been used clinically as an antibacterial agent for decades. In neonatal care, Triple dye, a combination of brilliant green, proflavine hemisulfate, and gentian violet is routinely used as a topical antibacterial agent on the umbilical stump of newborn babies [27], with a recent review of the practice categorizing toxicity as rare [28]. The concentrations of proflavine solution required for successful imaging (0.01–0.05%) [26] are substantially lower than that of the proflavine component in commercial triple dye, 0.11% (w/v) (VistaPharm, Kerr Triple Dye). The quantity of solution required for diagnostic imaging is approximately the same as that used in neonatal care (0.65 ml per single-use swab). The additional exposure to light which will occur during imaging can also be compared to that received by newborn babies undergoing phototherapy for jaundice. The high-resolution fiber-optic microendoscope proposed for use here delivers 0.5 mW of 455 nm light to the tissue through a 0.8 mm diameter fiber-optic bundle, corresponding to an irradiance level of 100 mW/cm^2^. The American Academy of Pediatrics defines intensive phototherapy as a spectral irradiance of at least 30 W/cm^2^ per nanometer over the 430–490 nm spectral band, equivalent to a total irradiance of 1.8 mW/cm^2^ [29]. Although the irradiance level is over 50-times higher with the fiber microendoscope system, a typical imaging session of 30 minutes (including imaging for routine care) is approximately 50-times shorter than a typical 24 hour (1440 minutes) phototherapy incubation, leading to an equivalent light dose in each scenario. Proflavine has also been safely used in previous clinical studies evaluating its effect as a photosensitizing agent for the treatment of genital herpes simplex virus [30]. Precancers of the epithelium are associated with a variety of morphological changes including increased nuclear size, pleomorphism, nuclear hyperchromasia and increased N/C ratio [31]. Clinical pathologists currently assess these features qualitatively, by examining stained sections at low and high power magnification. Recently, a number of studies have shown that quantitative analysis of digital images of stained histologic sections can aid in the identification of precancers [32]. In quantitative pathology, measurements of morphological and architectural features are used to make the diagnosis. In both cases, the morphologic information is acquired from stained tissue sections taken from biopsies, which are invasive, expensive, and painful and limit the area at risk which can be examined. In vivo microscopy provides an alternative approach to assess these morphologic changes in vivo. For example, nuclear morphometry acquired from two dimensional confocal images of the cervical epithelium can be used to distinguish normal epithelium from high grade dysplasia in the cervix [33].

As high resolution in vivo imaging systems such as the one presented here achieve more widespread clinical application, there is a growing need for image atlases to help clinicians and pathologists interpret these types of images. Such tools are beginning to be developed. For example, the Digital Atlas of Video Endoscopy (DAVE) is an endoscopic education tool containing both high-resolution images and videos of different gastrointestinal procedures and disorders. Developed in collaboration with the American Society of Gastrointestinal Endoscopy (ASGE), the largest endoscopic society worldwide, the DAVE project serves as an initial and free platform for dissemination to the public [34].

Modern digital cameras are also capable of on-board image processing. Pathologically relevant morphologic markers including nuclear size, distribution, and N/C ratio could potentially be extracted from images using relatively straightforward analysis algorithms. This information could be used to assist non-expert medical personnel in reaching a diagnosis, by providing quantitative evaluation of images in real-time. Alternatively, the same information could be used to guide the healthcare provider in biopsy site selection, by locating those sites with the highest suspicion for disease. The flash memory cards used for image storage are also widely compatible with cell phone and PDA hardware, enabling image transmission to remote experts in situations where further diagnostic interpretation is necessary.

A potential clinical application area for this type of cost-effective, compact imaging system may emerge in early cancer screening, particularly in developing countries. In the foreseeable future, the burden of cancer will continue to shift onto the populations of these regions, where early detection through screening programs offers the only opportunity to implement affordable treatment. Real-time diagnosis at the time of a clinical visit is also critical in reducing loss of patients to follow-up, and enables “see-and-treat” programs to be carried out. Several epithelial cancers such as those of the uterine cervix, oral cavity, and esophagus are significant contributors to mortality and morbidity in developing countries, and a high-resolution *in vivo* imaging system such as the one presented here could have significant impact on the management of these cancers.

In conclusion, we believe that recent advances in imaging performance coupled with declining costs will enable further development of clinically viable diagnostic imaging systems based upon consumer-grade electronics. Such instruments may find use in developing countries with limited technical and financial resources, or in industrialized nations with inefficient, overpriced healthcare systems.
